# Surfaces: a software to quantify and visualize interactions within and between proteins and ligands

**DOI:** 10.1093/bioinformatics/btad608

**Published:** 2023-10-03

**Authors:** Natália Teruel, Vinicius Magalhães Borges, Rafael Najmanovich

**Affiliations:** Department of Pharmacology and Physiology, Faculty of Medicine, Université de Montréal, Montreal H3T 1J4, Canada; Department of Biomedical Sciences, Joan C. Edwards School of Medicine, Marshall University, Huntington, WV, USA; Department of Pharmacology and Physiology, Faculty of Medicine, Université de Montréal, Montreal H3T 1J4, Canada

## Abstract

**Summary:**

Computational methods for the quantification and visualization of the relative contribution of molecular interactions to the stability of biomolecular structures and complexes are fundamental to understand, modulate and engineer biological processes. Here, we present Surfaces, an easy to use, fast and customizable software for quantification and visualization of molecular interactions based on the calculation of surface areas in contact. Surfaces calculations shows equivalent or better correlations with experimental data as computationally expensive methods based on molecular dynamics.

**Availability and implementation:**

All scripts are available at https://github.com/NRGLab/Surfaces. Surface’s documentation is available at https://surfaces-tutorial.readthedocs.io/en/latest/index.html.

## 1 Introduction

Molecular interactions determine all aspects of biological processes. Understanding the relative contributions of individual molecular interactions can guide the design of effective drugs to modulate biological processes as well as help understand the effect of mutations in natural processes or guide their introduction in protein engineering.

Protein engineering is an important tool in biotechnology that can produce proteins with improved therapeutic and industrial profiles (Chica 2015, Tobin *et al.* 2015, Bojar and Fussenegger 2020). In recent years, the field has made giant strides forward, but it still has the potential for exponential growth—as seen for many fields that benefit from high-throughput technologies and powerful new computational tools. However, a random search for all possible sequence configurations and respective structures and functions might reveal an enormous searchable space to explore. This is, even today, one of the biggest challenges of protein engineering, and one that makes the rational design necessary. Techniques that provide insights into the partial contribution of each atom or residue to protein interactions are critical for understanding the intricacies of the interaction interface. By identifying the specific residues and atoms that play a crucial role in the interaction, these techniques can aid in the development of rational protein engineering strategies. Unfortunately, these techniques, mostly based on *ab initio* approaches, are often computationally expensive, limiting the extent to which they can be used to explore this expansive searchable space (e.g. through simulating deep mutational scans or in large datasets).

With the decreasing cost of computational power, it is possible to simulate biological systems considering more realistically the underlying physical processes at the core of molecular interactions. Molecular dynamics (MD) simulations are at the forefront of such efforts, but such methods are computationally expensive and often also difficult to implement and thus remain impractical for high-throughput applications or broad adoption. Among these methods, we can highlight well known methods for modeling and analysis of protein structures, FoldX (Buß *et al.* 2018) and Rosetta (Kortemme *et al.* 2003, Leaver-Fay *et al.* 2011), free-energy perturbation (FEP) methods (McCarrick and Kollman 1999, Zhu *et al.* 2022, Sergeeva *et al.* 2023), molecular mechanics (MM/PBSA and MM/GBSA) methods (Genheden and Ryde 2015) that aim at predicting the ΔΔG of binding for different mutations relative to wild-type or the binding ΔG for biomolecular complexes, and gRINN (Serçinoglu and Ozbek 2018), a tool to breakdown the per-residue energetic contribution of molecular interactions based on MD simulations.

To address this challenge, simplified techniques using atomic surface areas in contact to estimate binding energy can be employed. This approach has previously been introduced in methods such as LPC (Ligand-Protein Contacts) and CSU (Contacts of Structural Units) (Sobolev *et al.* 1999) as well as STC (Structure-based Thermodynamic Calculations) (Lavigne *et al.* 2000). While these techniques are currently limited by server availability, as well as over-simplified atom type definitions and energetic pairwise matrices, they provide a faster alternative to evaluating energetic decompositions and served as the basis upon which many other methods that use atomic surfaces areas in contact were built (Gaudreault and Najmanovich 2015, Frappier *et al.* 2017, Ribeiro *et al.* 2019, Olechnovič and Venclovas 2021). Therefore, it is worth revisiting and improving these techniques by employing new libraries and visualization tools, making them customizable and user-friendly to broaden their general applicability. By doing so, we can enhance our understanding of the per-residue contribution to protein interactions and facilitate more efficient and effective protein engineering.

In this application note, we present Surfaces, a fast method that utilizes atomic surface areas in contact, user-defined atom-type definitions, and pairwise pseudo-energetic matrices as a proxy for highlighting favorable and unfavorable interactions within and between proteins, as well as between proteins and other biomolecules.

## 2 Methods

**Figure 1.**
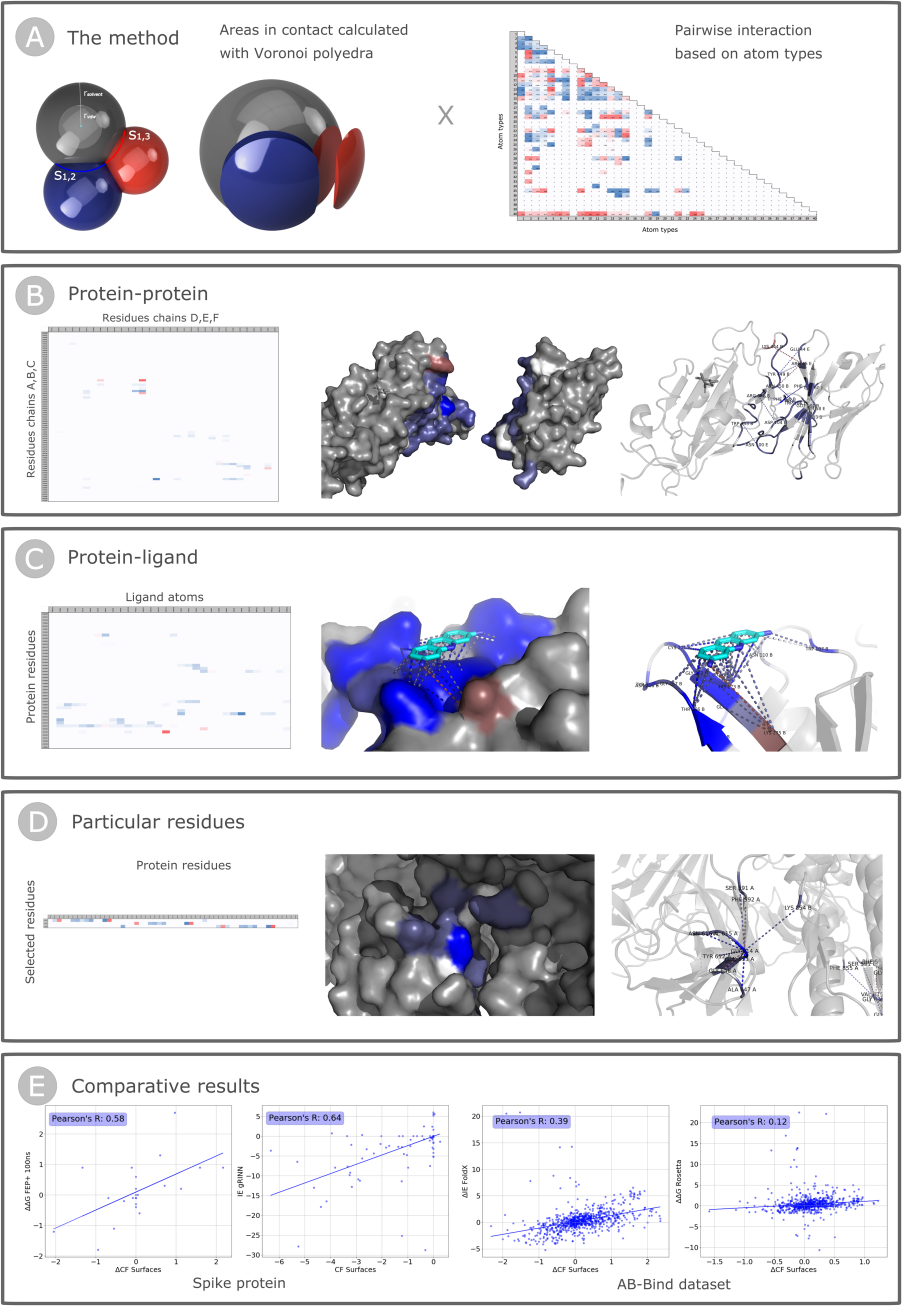
Schematic representation of the method and applications of Surfaces scripts. (A) The method: Representation of the calculated areas in contact between atoms 1 (gray), 2 (blue), and 3 (red), considering the expanded radii—that accounts for the van der Waals radii ($r_{\mathrm{vdw}}$) and the radius of a water molecule ($r_{\mathrm{solvent}}$)—and leading to the definition of the surfaces in contact $S_{1,2}$ and $S_{1,3}$, represented as exploded spherical caps, and the default matrix of pairwise interactions based on 40 SYBYL atom types. (B) Protein–protein: Example of the *protein–protein* application using the PDB structure 7VQ0 (Maeda *et al.* 2022). Representation of the. CSV output and of the visual output colored from red (unfavorable) to blue (favorable) showing the net value of interactions mapped into the surface of the chains, as well as the most relevant interactions highlighted with dash lines. (C) Protein–ligand: Example of the *protein–ligand* application using the PDB structure 7NT4 (Napolitano *et al.* 2022). Representation of the. CSV output and of the visual output colored from red (unfavorable) to blue (favorable) showing the net value of interactions mapped into the surface of the protein, as well as the interactions with the atoms of the ligand highlighted with dash lines. (D) Particular residues: Example of the *particular residue*s application using the PDB structure 7EAZ (Yang *et al.* 2021) and selecting the residues GLY614. Representation of the. CSV output and of the visual output colored from red (unfavorable) to blue (favorable) showing the net value of interactions mapped into the surface of the protein, as well as the interactions involving the residues of interest highlighted with dash lines. (E) Comparative results of Surfaces with the calculations performed using FEP + 100 ns MD simulations for the interaction of 23 mutants of the Spike protein and the receptor ACE2 (Sergeeva *et al.* 2023), and with the pairwise Interaction Energies calculated with gRINN (Serçinoglu and Ozbek 2018) for a MD simulation of the Spike protein Receptor-Binding Domain of the Delta variant and the receptor ACE2 (Cheng *et al.* 2022), as well as the comparison with published results obtained using FoldX (Schymkowitz *et al.* 2005) and Rosetta (Kortemme *et al.* 2003) for the prediction of the effects on binding of 1101 mutations that constitute the AB-Bind dataset (Sirin *et al.* 2016).

Surfaces quantifies atomic interactions using two measures. The first measure is the area in contact between atoms as described and calculated by Vcontacts using a Voronoi procedure (McConkey *et al.* 2002). This method restricts the evaluation of interactions to atoms within close proximity. The second measure is a pairwise pseudo-energetic matrix that assigns an interaction pseudo-energy based on the atom types (Fig. 1A).


(1) $${\mathrm{CF}}_{\mathrm{unit}1,\mathrm{unit}2}=\;\sum_{i=1}^{N}\sum_{j=1}^{M}\varepsilon_{ij}\times \;S_{ij}.$$

The complementarity function (CF) that accounts for these two measures is described by Equation (1), in which *N* is the total of atoms of unit 1, *M* is the total of atoms of unit 2, $\varepsilon_{ij}$ is the pseudo-energy for the interaction between atoms *i* and *j*, and $S_{ij}$ is the surface area in contact between atoms *i* and *j*. Units can correspond to residues, ligands or ligand atoms.

The CF calculation is based on the scoring function utilized by FlexAID (Gaudreault and Najmanovich 2015), a ligand docking tool, that also provides the SYBYL definition of atom types and the respective matrix of pairwise interaction energies optimized for the prediction of ligand poses using the PDBbind dataset (Wang *et al.* 2005). The 40 SYBYL atom types and the respective pairwise atom-type pseudo-energetic matrix from FlexAID are used as the default in Surfaces scripts. However, the Surfaces scripts are designed to be customizable, enabling the incorporation of modified residues or ligands, as well as the use of alternative atom type classifications and energetic matrices.

To visually represent the results of interaction analysis, Surfaces offers functions created using the PyMOL API (pymol.org). These functions are also customizable and allow for the visualization of the overall contribution of residues to interaction surfaces, as well as specific interactions between structural units, as PyMOL sessions.

## 3 Results and discussion

Surfaces was built as a set of python scripts designed to evaluate various types of interactions in proteins. The scripts are user-friendly, fast, and can be easily customized and automated, offering a scriptable way of generating data and visual representations for the interactions of interest.

### 3.1 Applications of surfaces

The specific types of interactions that can be analyzed by Surfaces are protein-protein, protein-ligand and residue interactions. In all cases, the input is a .PDB formatted file while the output of the quantitative analysis is saved as a .CSV file. The output for visualization is saved as a .PSE PyMOL session file. The visual output shows the surfaces of the relevant units of interest colored according to the net value of interactions and the specific pairwise interactions to the net total are represented as colored dash lines with a customizable color scale. In all cases, the input .PDB structure needs to be cleaned of any non-defined atoms in a pre-processing step, which can be done using scripts that are provided with the Surfaces software. The pre-processing is not done automatically to offer the use the possibility to customize. For example, if the user wishes to ignore specific hetero-atoms, these need to be removed but otherwise, defined. Surfaces offers a general script for atom type assignment mapping element types to the default SYBYL 40 atom types.

*Protein–protein interfaces*: For this application, two groups of protein chains are required as input, Surfaces calculates the interactions between residues of the first group and residues of the second group (Fig. 1B). This can also be used for protein–ligand evaluations by assigning a chain ID for the ligand’s atoms, if the user intends to evaluate the complete interaction of the ligand with each residue without a per-atom decomposition (see next paragraph).

*Protein–ligand interactions*: This application requires the PDB assigned three-letter code of the ligand of interest as input and calculates the interaction between residues of the structure and each atom of every instance of the ligand within the structure (Fig. 1C). Before running the application, two pre-processing steps are required: first, the assignment of atom types to the atoms of the ligand into the .DEF file, and second, cleaning the input .PDB structure of any non-defined atoms.

*Particular residue interactions*: This application requires a list of residues of interest as input and calculates all interactions (inter- or intra-chain) involving those residues (Fig. 1D).

### 3.2 Validation of surfaces

Common methods for analyzing protein-protein interactions and the per-residue energetic decomposition of the contributions of such interactions to the overall free energy are based on MD simulations (Kollman *et al.* 2000, Homeyer and Gohlke 2012, Serçinoglu and Ozbek 2018). MD simulations can provide detailed information on the structural changes and energetics associated with protein-protein interactions, including the binding free energy and per-residue energetic contributions. However, MD simulations are computationally intensive (Bopp *et al.* 2008, Ciccotti *et al.* 2022) making them less suitable for large-scale studies including protein engineering. Furthermore, MD simulations require expert knowledge to set up, which creates an additional obstacle for broad utilization.

The examples in Fig. 1 to illustrate the utilization of Surfaces to the three applications of Surfaces were taken from different datasets. The *protein-protein* interface analyzed in Fig. 1A was taken from a dataset of 738 structures of one or more chains of the SARS-CoV-2 Spike protein in complex with one or more chains of antibodies (Gowthaman *et al.* 2020). Such analysis required an average of 11.38 ± 9.62 CPU-seconds per structure including the time required for the preprocessing step. The analysis of *protein-ligand* interactions is derived from a dataset of 709 non-redundant structures and 669 non-redundant ligands, totaling 831 experimentally solved protein-ligand complexes of SARS-CoV-2 proteins and small molecules (Harrison *et al.* 2021). This analysis required an average of 3.97 ± 2.32 CPU-seconds per structure (including pre-processing).

To demonstrate the reliability of Surfaces, we performed two validations. The first one was performed using the AB-Bind dataset (Sirin *et al.* 2016), comprising 1101 mutations in 32 different protein complexes and associated experimentally determined ΔΔG changes. This dataset was published along with an extensive evaluation of the performance of seven different binding affinity prediction methods, performed using as input mutant structures generated with three different modeling methodologies (Supplementary Fig. S1A). By modeling the structures with two of these methods, FoldX (Buß *et al.* 2018) and Rosetta (Kortemme *et al.* 2003, Leaver-Fay *et al.* 2011), we can decouple the modeling quality from the prediction ability. Irrespective of the modelling method, Surfaces performance is similar to that of the best performing methods (Supplementary Fig. S1). Specifically, using FoldX mutants, Surfaces’ results showed a Pearson’s R of 0.32 and AUC |ΔΔG| > 0 of 0.65, compared to respective values of 0.34 and 0.70 obtained through FoldX for binding evaluation. For structures modelled with Rosetta, Surfaces predictions exhibited a Pearson’s R of 0.14, marginally lower than the correlation of 0.16 observed during interface assessment with Rosetta (Supplementary Fig. S1). Nonetheless, Surfaces shows slightly superior performance in ROC curve analysis compared to Rosetta, with an AUC for |ΔΔG| > 0 value of 0.61 compared to Rosetta’s 0.60 (Supplementary Fig. S1) (Detailed data available in the supplementary material). Other methods with equivalent or inferior performance to Surfaces are bASA (Hubbard and Thornton 1993, Sirin *et al.* 2016), dDFIRE (Yang and Zhou 2008), DFIRE (Zhou and Zhou 2002), and STATIUM (DeBartolo *et al.* 2012, 2014). Discovery Studio (Spassov and Yan 2013) shows slightly superior performance with Pearson’s R of 0.45 but in this as a paid software, we cannot test it to determine if the additional performance is due to a more accurate modelling of mutants or more accurate calculation of ΔΔG (Supplementary Fig. S1).

A second validation involved the comparison of binding affinity prediction performance for a highly curated dataset of 23 SARS-CoV-2 Spike Receptor Binding Domain (RBD)/ACE2 binding ΔΔG values measured by Surface Plasmon Resonance (Sergeeva *et al.* 2023). Surfaces shows a Pearson’s correlation of 0.556 with the experimental data, comparable to the performance of FEP + 100 ns MD, which obtains a correlation of 0.598, and considerably higher than all other methods tested, including state-of-the-art techniques such as MM/PBSA as MM/GBSA (Table S1 and Supplementary Fig. S2). Surfaces and FEP + 100 ns also obtained very similar ΔΔG root mean square errors (RMSE) and Pearson’s phi, showing an equivalent ability to classify mutations as stabilizing or destabilizing (detailed data available in the Supplementary Material).

Lastly, gRINN (Serçinoglu and Ozbek 2018) is a widely used method for residue-based energy decomposition that uses MD trajectories to calculate binding energy means and distributions. We used gRINN to calculate interface interactions of the Delta SARS-CoV-2 Spike in complex with the receptor ACE2 (Cheng *et al.* 2022) with MD trajectories available at the COVID-19 Molecular Structure and Therapeutics Hub (covid.molssi.org). The Pearson’s R obtained comparing Surfaces and gRINN is 0.64 (Fig. 1E and Supplementary Fig. S3). As additional validation of Surfaces (and comparison with gRINN) with experimental validation of per-residue contributions to binding, the two software were used to guide the selection of mutations to disrupt the binding interface between the Ebola protein VP35 and Ubiquitin (Rodríguez-Salazar *et al.* 2023). The two software highlighted a particular residue among the top contributing residues to the protein–protein interaction interface. Mutating this residue disrupted the interaction (Rodríguez-Salazar *et al.* 2023). The analysis of binding energy decomposition can help analyze interactions between proteins and ligands, potentially as a guide in rational drug design drug design (Supplementary Fig. S4).

In order to consider structural variations, captured when using MD-based approaches, Surfaces can be used to analyze protein ensembles, generated with tools such as the NRGTEN package (Mailhot and Najmanovich 2021). Such a procedure would permit to detect transient interactions utilizing the ensemble as a sample of the partition function near equilibrium by associating probabilities for each interaction.

## 4 Conclusions

Surfaces provides a simple, fast, and easy to use method to analyze and visualize biomolecular interactions. Surfaces performs equivalently or better than widely used methods such as Rosetta, FoldX, MM/GBSA and MM/PBSA, and, for specific datasets, comparable even to computationally expensive and cumbersome to implement methods such as gRINN and FEP. The use of variations in solvent accessible surface areas or those in contact to estimate free-energy contributions has been established previously (with LPC/CSU, STC) but somehow fell out of use over the past 20 years within the broader community. However, this work shows that such calculation can provide data of the same quality as more computationally expensive methods based on molecular dynamics, currently available methods based on machine learning, or other mean-field based statistical approaches.

As far as small-molecule protein interactions are concerned, docking simulations can be used to predict the binding pose, the relative ranking of molecules in virtual screening and the actual binding affinity. Until recently, no docking scoring function was able to tackle all three applications at once (Huang *et al.* 2010) and we believe this is still the case. The CF function parameters used here in the validations were optimized for the accurate detection of docking poses and tested for the relative ranking of ligands in virtual screening (Gaudreault and Najmanovich 2015). The CF function was not tested for the prediction of small-molecule binding affinities. As such, we do not believe Surfaces results correlate highly with binding affinities for small-molecules but this remains to be tested. Therefore, we recommend that Surfaces be used only to detect frustrated interactions between ligand and protein atom to guide rational drug design.

The scriptable and customizable nature of Surfaces makes it a valuable tool for researchers seeking to analyze large structural datasets, such as in virtual screening or protein engineering, thus permitting a broader exploration of search space. Lastly, the ease of use of Surfaces and low demand on computational resources makes this type of analysis accessible to a broader audience.

## Supplementary Material

btad608_Supplementary_Data

## Data Availability

All data is available in the supplementary data file and within the documentation site.
