# Supplementary material for: Surfaces: a software to quantify and visualize interactions within and between proteins and ligands
Source: Bioinformatics. 2023 Oct 3;39(10):btad608. doi: 10.1093/bioinformatics/btad608 (PMC10568369; doi:10.1093/bioinformatics/btad608)
Supplement: btad608_Supplementary_Data [file btad608_supplementary_data.pdf]

Supplementary materials

Surfaces: A software to quantify and visualize interactions within and between proteins and ligands

Natália Teruel[1], Vinicius Magalhães Borges[2] and Rafael Najmanovich[1,*]

[1]Department of Pharmacology and Physiology, Faculty of Medicine, Université de Montréal, Montreal, Canada

[2]Department of Biomedical Sciences, Joan C. Edwards School of Medicine, Marshall University, Huntington, WV, USA.

*To whom correspondence should be addressed.

Estimation of ΔΔG of binding

The antibody binding data (AB-Bind) dataset (Sirin *et al.*, 2016) contains 1101 mutants with experimentally determined changes in binding free energies (ΔΔG) across 32 antibody-antigen complexes and includes wild-type structures for each complex. Using the AB-Bind dataset, the authors compared the performance of protein scoring potentials in predicting changes in binding free energies differences as a result of mutagenesis. They evaluated the methods bASA (Sirin *et al.*, 2016; Hubbard and Thornton, 1993) dDFIRE (Yang and Zhou, 2008), DFIRE (Zhou and Zhou, 2002), FoldX (Buß *et al.*, 2018), Rosetta (Kortemme *et al.*, 2003), Discovery Studio (Spassov and Yan, 2013) and STATIUM (DeBartolo *et al.*, 2012, 2014). Their detailed data allows us to compare the performance of Surfaces against experimental results of these 32 complexes and 1101 mutations relative to the above methods.

We performed all the calculations on mutants modeled on the same crystal structures following the same methodology as Sirin *et al.* employing as marked either FoldX or Rosetta for the most direct comparison with the results presented (Sirin *et al.*, 2016). Surface's CF scoring for the full binding interface was calculated by summing all individual interaction contributions from the per-residue outputs.

The CF values generated by Surfaces are based on a pseudo-physical model and need to be rescaled to be compared to the experimental results in kcal/mol. In order to classify the results into

the three tiers of mutations used for the ROC curve evaluations we performed a to a linear regression to the experimental values Surfaces results without any loss of generalization, as a linear transformation does not affect the relative ranking of results.

The statistical metrics originally employed to compare results across methods were the Pearson's correlation coefficient (Pearson's R) and the area under the ROC curve (AUC). We applied these same metrics to evaluate Surfaces' results, presented in Figure S1. In Figure S1A, we depict the outcomes obtained by the various protein scoring potential methods aforementioned, based on Sarin *et al*. In Figure S1B we plot Surfaces results for the same dataset. These predictions produced different results for mutant structures generated via FoldX and Rosetta. For Pearson's R and AUC for different confidence levels ([ΔΔG] > 0, [ΔΔG] > 0.5, and [ΔΔG] > 1), Surfaces (scoring FoldX-generated mutants) outperforms bASA, dDFIRE, and DFIRE. Moreover, Surfaces' results exhibit a modestly comparable accuracy to the top-performing method for this specific set of mutant structures, FoldX. In comparison to Rosetta, Surfaces (scoring Rosetta-generated mutants) achieves slightly lower Pearson's R but superior AUC across all scenarios. Among all the methods, only STATIUM doesn't require input structures of the mutated complexes, and demonstrates nearly equivalent performance to Surfaces (scoring FoldX-generated mutants), with matching Pearson's R and subtle variations in AUC. Due to the proprietary nature of Discovery Studio, a comprehensive comparison between Discovery Studio and Surfaces, both scoring Discovery Studio-generated mutants, isn't feasible, hindering the generation of mutants for assessment.

A

	Foldx mutants			
	**bASA**	**dDFIRE**	**DFIRE**	**FoldX**
**Pearson's R**	0.22	0.19	0.31	0.34
**AUC |DDG| > 0**	0.63 (0.59 - 0.66)	0.59 (0.56 - 0.63)	0.65 (0.62 - 0.69)	0.70 (0.67 - 0.73)
**AUC |DDG| > 0.5**	0.67 (0.63 - 0.73)	0.63 (0.58 - 0.67)	0.73 (0.69 - 0.77)	0.81 (0.78 - 0.85)
**AUC |DDG| > 1.0**	0.68 (0.63 - 0.75)	0.66 (0.61 - 0.72)	0.78 (0.74 - 0.83)	0.87 (0.83 - 0.91)

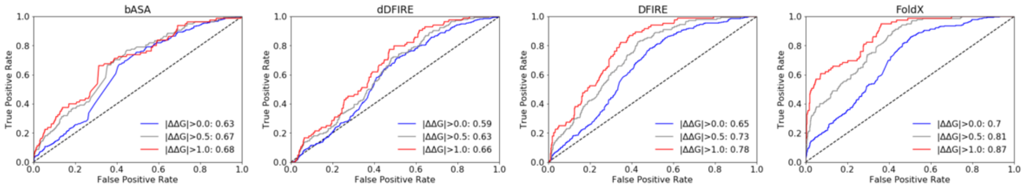

	Rosetta mutants		Discovery Studio mutants
	**Rosetta**	**STATIUM**	**Discovery Studio**
**Pearson's R**	0.16	0.32	0.45
**AUC |DDG| > 0**	0.60 (0.57 - 0.64)	0.64 (0.60 - 0.67)	0.73 (0.70 - 0.76)
**AUC |DDG| > 0.5**	0.64 (0.60 - 0.69)	0.74 (0.70 - 0.78)	0.82 (0.78 - 0.86)
**AUC |DDG| > 1.0**	0.70 (0.64 - 0.75)	0.81 (0.76 - 0.85)	0.88 (0.85 - 0.92)

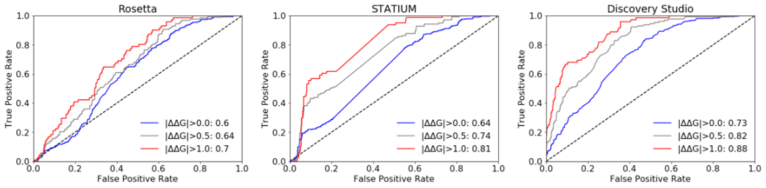

B

	Foldx mutants	Rosetta mutants
	**Surfaces**	**Surfaces**
**Pearson's R**	0.32	0.14
**AUC |DDG| > 0**	0.65 (0.61 - 0.68)	0.61 (0.58 - 0.65)
**AUC |DDG| > 0.5**	0.74 (0.69 - 0.78)	0.68 (0.64 - 0.73)
**AUC |DDG| > 1.0**	0.79 (0.73 - 0.84)	0.73 (0.68 - 0.79)

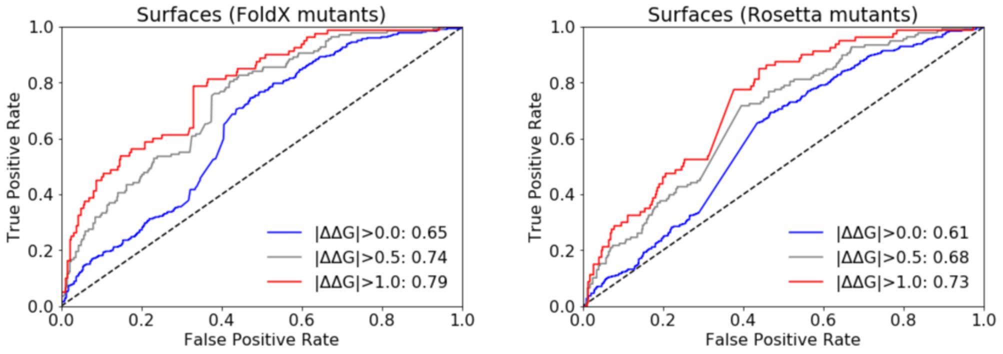

Fig S1. Comparative evaluation of different binding affinity prediction methods applied to the AB-Bind dataset. (A) From Sarin et al. (Sirin *et al.*, 2016) we have the quantitative correlations (Pearson's correlation coefficient, Pearson's R) and the area under the ROC curve (AUC) presented for the entire dataset in blue, medium confidence subset (|ΔΔG| > 0.5 kcal/mol) in gray, and high confidence subset (|ΔΔG| > 1 kcal/mol) in red. Results are from predictions performed using the scoring functions bASA, dDFIRE, DFIRE and FoldX (using mutants generated by FoldX), Rosetta (with mutants generated by Rosetta), Discovery Studio (using mutants generated by Discovery Studio) and STATIUM; **(B)** Surfaces results for the same dataset with quantitative correlations (Pearson's correlation coefficient, Pearson's R)

and the area under the ROC curve (AUC), with binding predictions performed to mutant structures generated using FoldX and Rosetta.

The second method validation was based on the work by Sergeeva *et al.* (Sergeeva *et al.*, 2023), that compares binding prediction results using free-energy pertubations (FEP) against a broad range of different methods, specifically methods based on machine-learning: Mutabind2 (Zhang *et al.*, 2020), mCSM-PPI (Rodrigues *et al.*, 2019), SAAMBE-3D (Pahari *et al.*, 2020); based on statistical potentials: BeAtMusic (Dehouck *et al.*, 2013); and force field related scoring functions: FoldX (Schymkowitz *et al.*, 2005), Rosetta flex ddG (Barlow *et al.*, 2018)Click or tap here to enter text.; and molecular mechanics: MM/PBSA and MM/GBSA (Singh and Warshel, 2010; Genheden and Ryde, 2015). The authors compare the performance of these methods on a set of SARS-CoV-2 Spike mutations for which they have experimentally determined binding affinity to the receptor ACE2 using surface plasmon resonance (Pattnaik, 2005). Their data allows us to compare the performance of Surfaces to that of the various methods against experimental results for the evaluation of this particular interaction.

To do so, we performed all the calculations on mutants modeled to the same crystal structure of ACE2/RBD (PDB 6M0J) (Lan *et al.*, 2020), following the same methodology used by Sergeeva *et al.* (Sergeeva *et al.*, 2023). In order to do the RMSE calculation, as well as to follow particular numerical thresholds used for the analysis of the data, we also produced Surfaces results subjected to a linear regression to the experimental values.

The statistical metrics originally used to compare the results using the different methods were the Pearson's correlation coefficient (Pearson's R), root mean square error (RMSE) and Pearson's phi for stabilizing mutations, performing a binary classification of the data, considering as stabilizing the mutations with $\Delta\Delta G \leq -0.4$ (Pearson's Φ (stabilizing ≤ -0.4)), a threshold defined by Sergeeva *et al.* based on the experimental accuracy (Sergeeva *et al.*, 2023). We utilized the same statistical metrics to Surface results. The results are shown in Table S1. In Figure S1A we plot the experimental vs. calculated $\Delta\Delta G$ values for all the methods presented by Sergeeva *et al.* and in Figure S1B for Surfaces. According to all metrics, Surfaces' performance is equivalent to that of FEP, with a superior Pearson's R to all other methods at a fraction of the computational cost.

It is worth noting that Surfaces provides a simplified estimation of changes in enthalpy differences ($\Delta\Delta H$), not of the free energy. In the past we have utilized our ENCoM normal mode

analysis method to estimate ΔΔG of mutations based solely on vibrational entropy differences (Frappier and R. J. Najmanovich, 2014; Frappier and R. Najmanovich, 2014). Such vibrational entropy differences, as calculated for a large number of Spike mutants (Teruel *et al.*, 2021), can be performed in a straightforward manner with NRGTEN package (Mailhot and Najmanovich, 2021) and combined with Surfaces ΔΔH predictions.

According to the different metrics used to compare predictive performance, FEP calculations based on a 100ns Molecular Dynamics (MD) trajectories are shown to represent a good alternative (Table S1). As noted by the authors (Sergeeva *et al.*, 2023), being MD-based, FEP calculations require a complex computational infrastructure and are time consuming. Surfaces, presenting a close Pearson's correlation, similar error compared to the experimental values, as well as a comparable classification performance, measured by the Pearson's phi for the threshold value used for classifying a mutation as "stabilizing" (Table S1, Fig S1), required a very small fraction of the computational power to evaluate all mutants (185 CPU-seconds). The results from Table S1 and Fig S2 were generated with the goal to understand binding affinities of mutations and variants already selected during viral evolution. However, some of these same methods, with significant computational cost, would not be suitable for exploratory objectives while Surfaces can be used in high-throughput computational mutational scans.

Table S1. Comparative measures of binding affinity of mutants of the SARS-CoV-2 RBD and the receptor ACE2. From (Sergeeva *et al.*, 2023) we have the experimental values (ΔΔG experiment SPR), as well as the predictions using different binding affinity calculation methods (left table) and the analysis of Pearson's correlation coefficient (Pearson's R), root mean square error (RMSE) and Pearson's phi for stabilizing mutations (Pearson's Φ (stabilizing ≤ -0.4)) (lines in gray). Added to this table, ΔCF Surface results calculated for the same mutants, the linear regression of these values to the experimental reference (right table) and the statistical analysis for Surfaces results (light blue lines). Blue values correspond to stabilizing mutations with ΔΔG ≤ -0.4. Red values correspond to destabilizing mutations with ΔΔG ≥ 0.4. This table is also available in word format for easy of reuse as a supplementary Table S1 file.

RBD mutation	ΔΔG experiment SPR	ΔΔG Mutabind2	ΔΔG mCSM-PPI	ΔΔG SAAMBE-3D	ΔΔG BeAtMusic	ΔΔG FoldX	ΔΔG Rosetta flex	ΔΔG MM / PB-SA	ΔΔG MM / GB-SA	ΔΔG FEP+ 100ns	ΔCF Surfaces (linear regression)
N501Y	-0.8	0.7	0.5	0	0.1	6	0.4	5.8	2.6	-1.2	-2.0
Y453F	-0.7	-0.2	0.1	0.1	0.3	-0.4	-0.2	1.3	2.2	-0.6	0.1
S477N	-0.5	-0.1	-0.1	0.2	0.1	0	0.1	5.4	9.3	-0.1	-0.7
N501T	-0.5	-0.6	-0.9	-0.1	0.4	-0.9	-0.4	3.1	1.8	-1.8	-0.9
N439K	-0.1	-0.1	0.5	0.6	0.3	0	0	-8.8	-0.8	0.6	0.0
N440K	0	0.1	-0.1	0.5	0	-0.1	0	-1.9	-2.9	-0.4	0.0
F490S	0	0.6	0.2	0.8	0.7	0	0	-1	2.2	-0.1	-0.1
L452M	0	0	0.5	-0.3	0.2	0	0	6.3	2.1	0.2	0.0
L452R	0	-0.8	0	0.1	0.2	-0.3	0	-6.8	-3.8	-0.3	0.0
E484Q	0.1	0.1	0.3	0.5	0.2	-0.1	0	7.7	11.3	0.3	0.7
T478K	0.1	0.2	0	0.1	0.2	0	0.1	0.8	4.9	0	0.0
N481K	0.1	0	0	0.6	0.2	0	0	-13.2	-7.4	-0.1	0.0
E484K	0.1	0.2	0.3	0.3	0.1	-0.2	-0.1	3.1	7.2	0.2	1.1
Q498R	0.2	0.4	1.4	1.1	0.8	-0.5	-0.6	-1	-6.4	2.7	1.0
S477I	0.2	0.1	0	0	0.2	0.1	0	-1.3	6.1	0	0.0
G446V	0.2	-0.7	0.1	-0.3	1.3	0.1	0	6	6.6	0.9	-0.1
T478R	0.2	0.1	0	0.3	0.2	0	0	1.6	3.4	-0.1	0.0
S477R	0.3	0.2	-0.1	0	0.1	0	-0.2	0.6	3.1	-0.2	-0.3
A475V	0.3	0.9	0	0.1	0.1	1	0.2	10	12.2	-1.1	-0.5
L455F	0.4	1.5	-0.7	0.2	-0.1	5	-0.6	6.5	5.1	0.9	-1.3
K417T	0.4	0.2	0.4	0.5	0.5	0.8	0.7	11.4	14.1	0.9	2.2
F486L	0.6	0.3	0.9	0.5	0.7	1.1	1.8	5.9	7.8	1.3	0.6
K417N	0.6	0.6	0.5	0.4	0.5	0.8	0.9	12.2	13.1	0.9	1.6
Pearson's R		0.322	0.218	0.259	0.219	-0.079	0.344	0.205	0.301	0.598	0.556
RMSE		0.523	0.532	0.475	0.497	1.741	0.501	6.461	6.934	0.754	0.747
Pearson's Φ (stabilizing ≤ -0.4)		0.163	0.266	-	-	0.504	0.163	-0.303	-0.242	0.592	0.593

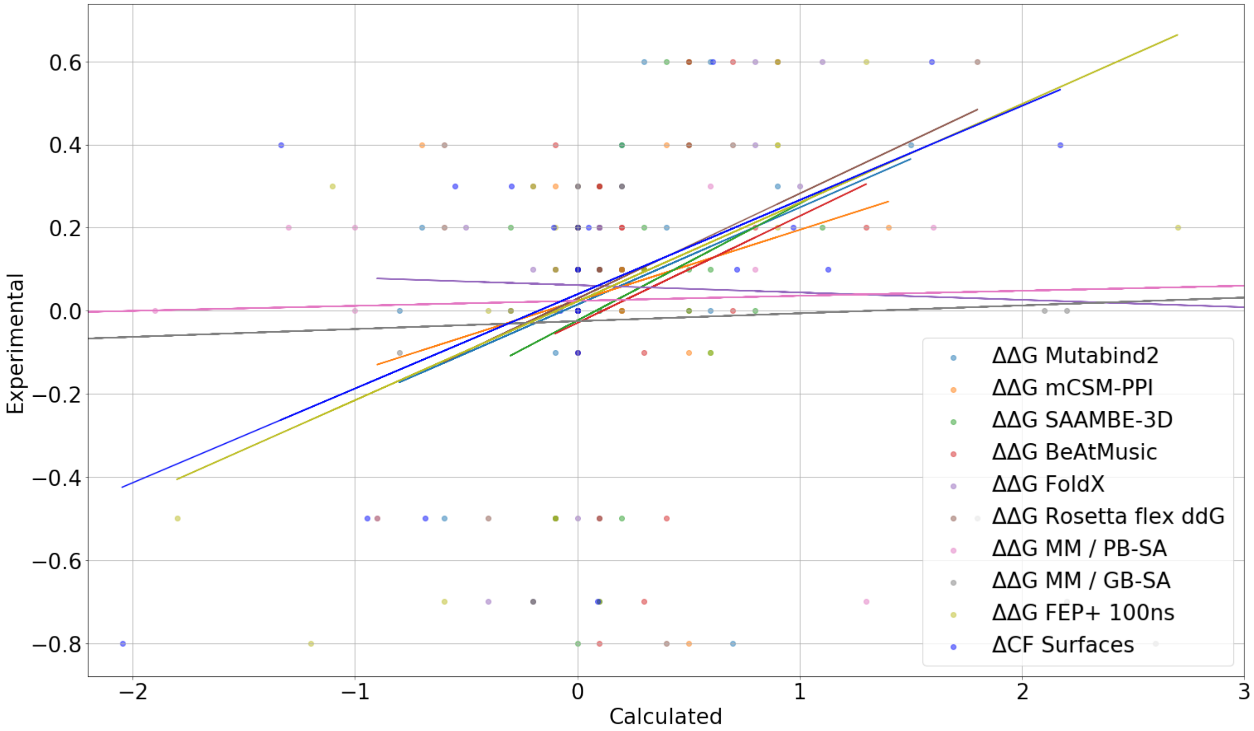

Fig S2. Graphic representation of the calculated binding affinities for each of the mutants against the experimental values, as well as their tendency lines. Values of ΔΔG predictions using different binding affinity calculation methods compared to experimental data (Sergeeva *et al.*, 2023). Linear regression of Surfaces' ΔCF prediction values for binding affinity compared to experimental data.

While the measure of full binding of mutants is often used to evaluate the impact of mutations on protein interactions, it does not always reflect the changes in interactions of the mutated residue alone, and may be associated with a disruption of adjacent regions in the interface. This point is also discussed when observing the non-cumulative effects seen for single mutants on full interfaces – the sum of the effects of individual mutations may not equal the effect of multiple mutations combined – as pointed out in the first evaluations of the Omicron Spike (Cameroni *et al.*, 2022; Dejnirattisai *et al.*, 2022).

Due to these limitations, for exploratory objectives and rational design, per-residue energetic decomposition methods offer very important information for purposes of protein engineering.

All data presented above, including individual predictions for each mutant are available in the Github repository for Surfaces: https://github.com/NRGlab/Surfaces.

Per-residue decomposition

The most common methods for analyzing protein-protein interactions and per-residue energetic decomposition are based on molecular dynamics (MD) simulations (Homeyer and Gohlke, 2012; Kollman *et al.*, 2000; Serçinoglu and Ozbek, 2018). MD simulations can provide detailed information on the structural changes and energetics associated with protein-protein interactions, including the binding free energy and per-residue energetic contributions. However, MD simulations are computationally intensive and therefore can take a long time to complete (Ciccotti *et al.*, 2022; Bopp *et al.*, 2008), making them less suitable for large-scale studies such as in computational protein design.

A widely used method for residue-based energy decomposition is gRINN (Serçinoglu and Ozbek, 2018), which uses MD trajectories to calculate binding energy means and distributions. From trajectories of the SARS-CoV-2 Spike Delta variant in complex with the receptor ACE2 (Cheng *et al.*, 2022), available at the COVID-19 Molecular Structure and Therapeutics Hub (MolSSI), we calculated interface interactions using gRINN and compare with Surfaces. We generated the results for gRINN considering only residues that were at a maximum distance of 6.8Å from the interface, a filtering distance of 20Å between residues and interactions present in 100% of the trajectory frames – these parameters reduced gRINN analysis to interactions in closer proximity, inter-chain interacting residues within the interface and filtered off transient interactions, to more closely match the interaction that are analyzed with Surfaces. Considering the net value of interactions for each residue, the Pearson's correlation coefficient between the results of the two methods is 0.634 (p= 6.07E-90) (Fig S2).

Whereas Surfaces does not detect transient interactions, it is possible to generate protein ensembles with the NRGTEN package (Mailhot and Najmanovich, 2021). Such an approach would make possible to generate a distribution of energies for each interaction. Furthermore, the possibility of calculating transition probabilities and occupancies for different configurations opens the possibility to apply statistical mechanics techniques.

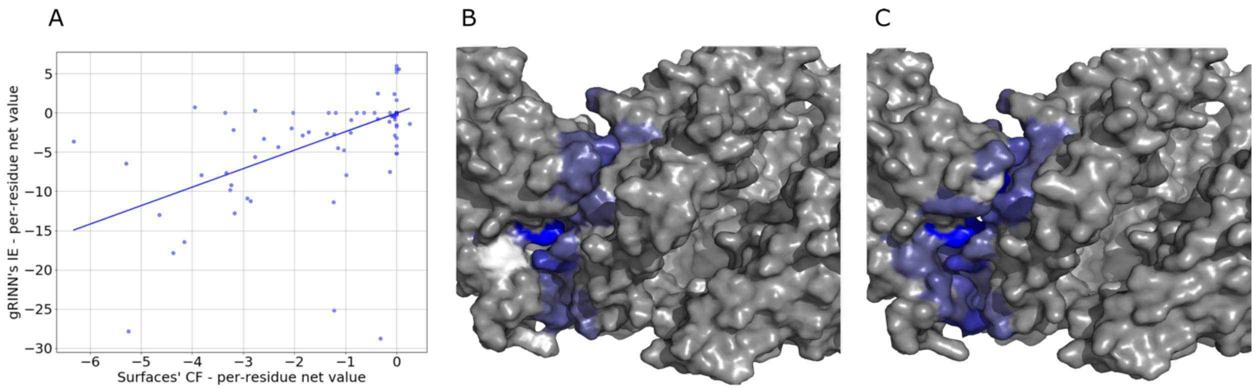

Fig S3. Per-residue result comparison between gRINN and Surfaces calculations. (A) Graphic representation of the calculated binding affinities for each residue of the complex using gRINN and Surfaces, as well as the tendency line of the correlation between the values. **(B)** Visual representation of the per-residue interactions according to gRINN results in a scale of dark blue (favorable) to white (neutral) generated with Surfaces visual output scripts. **(C)** Visual representation of the per-residue interactions according to Surfaces results in a scale of dark blue (favorable) to white (neutral) generated with Surfaces visual output scripts.

Ligand binding evaluation

The same rationale and scoring function can also be used to evaluate binding between a protein and a ligand, as well as the binding energy decomposition per residue of the protein and per atom of the ligand. Surfaces employs a scoring function first introduced by FlexAID (Gaudreault and Najmanovich, 2015), offering as default a matrix of pairwise interactions based on 40 SYBYL atom types that was optimized from the evaluation of ligand interactions of the PDBbind dataset (Wang *et al.*, 2005).

The concept of "frustration" in statistical physics refers to a system where a global energy minima cannot be reached by minimizing the energy of each interaction present in the system, some interactions remain at a state with higher energy than the lowest possible and are said to be frustrated. Biomolecules have been shown to harbor frustrated interactions (Bryngelson and Wolynes, 1987; Ferreiro *et al.*, 2011, 2013). In that context, Surfaces can be used to identify potential frustrated interactions not only between interacting amino acids as shown above, but also between ligands and proteins. The quantification and visualization of the energetic contribution of individual interactions within a specific ligand-protein or protein-protein complex with Surfaces helps to understand and exploit frustration in drug design and protein engineering.

As an example, we utilize Surfaces to visualize the interactions between ligand protein complexes from the BioLiP dataset (Yang *et al.*, 2013). First, two distinct proteins (DMSP lyase and Sigma-54 dependent transcriptional regulator) bound to the ATP (Fig S4 A,B and Table S2

A,B), and second, two similar molecules (ATP and GTP) bound to the same binding site (Sigma-54 dependent transcriptional regulator) (Fig S4 B,C and Table S2 B,C). Surfaces identifies frustrated interactions between ATP and DMSP lyase residues (residue ASP 721, figure S4A) as well as different frustrated interactions for Sigma-54 dependent transcriptional regulator (residue ASP 241, figure S4B), illustrating probable different convergent evolutionary paths. The comparison between interactions of ATP and GTP with the same Sigma-54 dependent transcriptional regulator binding site reveals visible changes in some interactions, namely in the strength of the favorable interaction with residue ARG 357 and the existence of interactions with ASP 241 and ILE 199, motivated by the small difference between the molecules and also by the different conformations of the ligands.

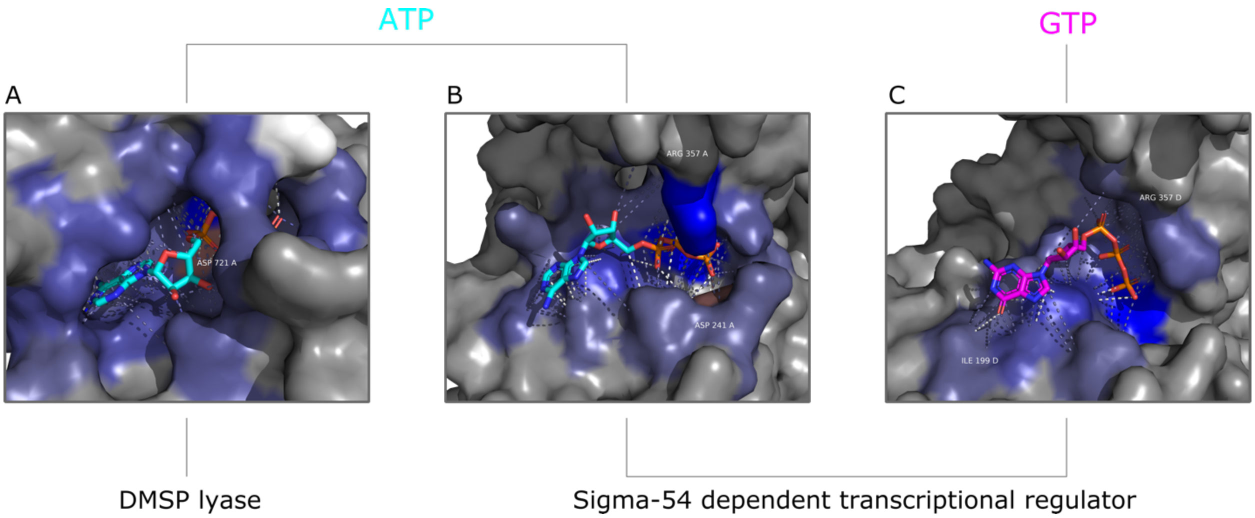

Fig S4. Illustration of the use of Surfaces for the assessment and visualization of protein-ligand interactions with the same molecule in different binding sites (A, B), and slightly different molecules in the same site (B, C). (A) ATP bound to DMSP lyase (PDB 7CM9), with the interacting residue ASP 721 highlighted for its unfavorable interaction; **(B)** ATP bound to Sigma-54 dependent transcriptional regulator (PDB 7V3W), with the interacting residue ASP 241 highlighted for its unfavorable interaction and residue ARG 357 highlighted for its particularly favorable interaction; and **(C)** GTP bound to Sigma-54 dependent transcriptional regulator (PDB 7V2B), with the interacting residue ARG 357 highlighted for its less favorable interaction, and residue ILE 199, highlighted for its new favorable interaction, both compared to those seen for ATP**.**

Table S2. Numeric results for ligand interactions. Results calculated for the interactions of ATP and DMSP lyase (PDB 7CM9), ATP and Sigma-54 dependent transcriptional regulator (PDB 7V3W), and GTP and Sigma-54 dependent transcriptional regulator (PDB 7V2B).

ATP - DMSP lyase

A	Adenine	Ribose	Phosphate Tail	Net value
HIS292A	0	0	-3183.872	-3183.872
PRO540A	-11.10447	0	0	-11.10447
VAL563A	38.9475	0	0	38.9475
LYS565A	906.6816	0	-2940.21424	-2033.5326
HIS571A	0	0	-1353.11244	-1353.1124
LYS572A	0	0	-6447.99838	-6447.9984
THR573A	0	-425.785	-2904.5722	-3330.3572
ASP574A	0	0	129.6379	129.6379
VAL578A	-241.1939	-563.6139	-811.88086	-1616.6887
LEU580A	-1854.1257	-1140.4004	0	-2994.5261
GLN606A	-2153.420838	0	0	-2153.4208
GLU607A	-1178.1372	0	0	-1178.1372
MET608A	-3524.905124	0	0	-3524.9051
VAL609A	-3403.856398	0	0	-3403.8564
THR610A	-14.1846	0	0	-14.1846
GLU614A	-35.568	-2522.6801	0	-2558.2481
GLY635A	0	-383.9729	233.63	-150.3429
ILE636A	0	-1783.9936	-480.6071	-2264.6007
THR638A	0	0	-16.6158	-16.6158
GLU706A	0	0	39.7496	39.7496
ASN708A	0	-22.3005	-2468.9372	-2491.2377
PRO709A	-151.3509	-1226.3133	-209.57164	-1587.2358
LEU720A	-3359.720676	-209.135	0	-3568.8557
ASP721A	-16.5864	0	1317.3463	1300.7599
LEU723A	0	0	-31.6931	-31.6931
**Net value**	-14998.52511	-8278.1947	-19128.71116	-42405.431

ATP - Sigma-54 dependent transcriptional regulator

B	Adenine	Ribose	Phosphate Tail	Net value
SER176A	0	0	-2284.54998	-2284.55
GLY177A	0	0	-3266.348	-3266.348
THR178A	0	0	-1094.181	-1094.181
GLY179A	0	0	-1572.6553	-1572.6553
LYS180A	0	0	-6123.3452	-6123.3452

GLU181A	-2210.2904	-1543.7587	-886.5917	-4640.6408
THR182A	0	-252.1202	0	-252.1202
LYS185A	-134.7645	0	0	-134.7645
PHE198A	-1828.7108	0	0	-1828.7108
SER200A	-22.4961	0	0	-22.4961
ASN202A	-57.2135	188.569	-1258.4065	-1127.051
ARG204A	0	0	-620.7089	-620.7089
ASN240A	0	0	-3271.67759	-3271.6776
ASP241A	0	0	1311.3744	1311.3744
SER280A	0	0	-688.7639	-688.7639
ARG357A	0	-111.1453	-6417.4291	-6528.5744
MET360A	0	-216.196	0	-216.196
**Net value**	-4253.4753	-1934.6512	-26173.28277	-32361.409

GTP - Sigma-54 dependent transcriptional regulator

C	Guanine	Ribose	Phosphate Tail	Net value
ILE147D	0	0	-92.6177	-92.6177
GLY177D	0	0	-1975.93371	-1975.9337
THR178D	0	0	-2916.6786	-2916.6786
GLY179D	0	0	-3507.65178	-3507.6518
LYS180D	0	0	-5289.05766	-5289.0577
GLU181D	-891.1528	-385.7914	-2512.3375	-3789.2817
THR182D	0	-175.5332	-2030.1703	-2205.7035
LYS185D	164.72743	-960.2644	0	-795.53697
PHE198D	-400.14345	0	0	-400.14345
ILE199D	-28.1288	0	0	-28.1288
SER200D	-2729.45	0	0	-2729.45
ASN202D	-188.5354	0	0	-188.5354
ASN240D	0	0	-1640.4008	-1640.4008
VAL356D	0	0	-931.40861	-931.40861
ARG357D	0	0	-1504.27755	-1504.2776
MET360D	0	-88.6728	-315.8504	-404.5232
**Net value**	-4072.68302	-1610.2618	-22716.38461	-28399.329

References

Barlow,K.A. *et al.* (2018) Flex ddG: Rosetta Ensemble-Based Estimation of Changes in Protein–Protein Binding Affinity upon Mutation. *J Phys Chem B*, **122**, 5389–5399.

Bopp,P.A. *et al.* (2008) SCOPE AND LIMITS OF MOLECULAR SIMULATIONS. *Chem Eng Commun*, **195**, 1437–1456.

Bryngelson,J.D. and Wolynes,P.G. (1987) Spin glasses and the statistical mechanics of protein folding. *Proc. Natl. Acad. Sci.*, **84**, 7524–7528.

Buß,O. *et al.* (2018) FoldX as Protein Engineering Tool: Better Than Random Based Approaches? *Computational and structural biotechnology journal*, **16**, 25–33.

Cameroni,E. *et al.* (2022) Broadly neutralizing antibodies overcome SARS-CoV-2 Omicron antigenic shift. *Nature*, **602**, 664–670.

Cheng,M.H. *et al.* (2022) Impact of new variants on SARS-CoV-2 infectivity and neutralization: A molecular assessment of the alterations in the spike-host protein interactions. *Iscience*, **25**, 103939.

Ciccotti,G. *et al.* (2022) Molecular simulations: past, present, and future (a Topical Issue in EPJB). *Eur. Phys. J. B*, **95**, 3.

DeBartolo,J. *et al.* (2014) Genome-Wide Prediction and Validation of Peptides That Bind Human Prosurvival Bcl-2 Proteins. *Plos Comput Biol*, **10**, e1003693.

DeBartolo,J. *et al.* (2012) Predictive Bcl-2 Family Binding Models Rooted in Experiment or Structure. *J Mol Biol*, **422**, 124–144.

Dehouck,Y. *et al.* (2013) BeAtMuSiC: prediction of changes in protein–protein binding affinity on mutations. *Nucleic Acids Res*, **41**, W333–W339.

Dejnirattisai,W. *et al.* (2022) SARS-CoV-2 Omicron-B.1.1.529 leads to widespread escape from neutralizing antibody responses. *Cell*, **185**, 467-484.e15.

Ferreiro,D.U. *et al.* (2013) Frustration in Biomolecules. *arXiv.org*, **q-bio.BM**.

Ferreiro,D.U. *et al.* (2011) On the role of frustration in the energy landscapes of allosteric proteins. *Proc. Natl. Acad. Sci.*, **108**, 3499–3503.

Frappier,V. and Najmanovich,R. (2014) Vibrational entropy differences between mesophile and thermophile proteins and their use in protein engineering. *Protein Sci*, **24**, 474–483.

Frappier,V. and Najmanovich,R.J. (2014) A Coarse-Grained Elastic Network Atom Contact Model and Its Use in the Simulation of Protein Dynamics and the Prediction of the Effect of Mutations. *PLoS Comput Biol*, **10**, e1003569.

Gaudreault,F. and Najmanovich,R.J. (2015) FlexAID: Revisiting Docking on Non-Native-Complex Structures. *J Chem Inf Model*, **55**, 1323–1336.

Genheden,S. and Ryde,U. (2015) The MM/PBSA and MM/GBSA methods to estimate ligand-binding affinities. *Expert Opinion On Drug Discovery*, **10**, 449–461.

Homeyer,N. and Gohlke,H. (2012) Free Energy Calculations by the Molecular Mechanics Poisson−Boltzmann Surface Area Method. *Mol Inform*, **31**, 114–122.

Huang,S.-Y. *et al.* (2010) Scoring functions and their evaluation methods for protein-ligand docking: recent advances and future directions. *Physical chemistry chemical physics : PCCP*, **12**, 12899–12908.

Hubbard,S. and Thornton,J. (1993) Naccess Dept. of Biochemistry and Molecular Biology, University College London.

Kollman,P.A. *et al.* (2000) Calculating Structures and Free Energies of Complex Molecules: Combining Molecular Mechanics and Continuum Models. *Accounts Chem Res*, **33**, 889–897.

Kortemme,T. *et al.* (2003) An orientation-dependent hydrogen bonding potential improves prediction of specificity and structure for proteins and protein-protein complexes. *Journal of molecular biology*, **326**, 1239–1259.

Lan,J. *et al.* (2020) Structure of the SARS-CoV-2 spike receptor-binding domain bound to the ACE2 receptor. *Nature*, **581**, 215–220.

Mailhot,O. and Najmanovich,R. (2021) The NRGTEN Python package: an extensible toolkit for coarse-grained normal mode analysis of proteins, nucleic acids, small molecules and their complexes. *Bioinformatics*, **37**, 3369–3371.

MolSSI COVID-19 Molecular Structure and Therapeutics Hub (https://covid.molssi.org).

Pahari,S. *et al.* (2020) SAAMBE-3D: Predicting Effect of Mutations on Protein–Protein Interactions. *Int J Mol Sci*, **21**, 2563.

Pattnaik,P. (2005) Surface plasmon resonance: applications in understanding receptor-ligand interaction. *Appl Biochem Biotechnol*, **126**, 79–92.

Rodrigues,C.H.M. *et al.* (2019) mCSM-PPI2: predicting the effects of mutations on protein–protein interactions. *Nucleic Acids Res*, **47**, W338–W344.

Schymkowitz,J. *et al.* (2005) The FoldX web server: an online force field. *Nucleic acids research*, **33**, W382-8.

Serçinoglu,O. and Ozbek,P. (2018) gRINN: a tool for calculation of residue interaction energies and protein energy network analysis of molecular dynamics simulations. *Nucleic acids research*, **46**, W554–W562.

Sergeeva,A.P. *et al.* (2023) Free Energy Perturbation Calculations of Mutation Effects on SARS-CoV-2 RBD::ACE2 Binding Affinity. *J. Mol. Biol.*, **435**, 168187.

Singh,N. and Warshel,A. (2010) Absolute binding free energy calculations: on the accuracy of computational scoring of protein-ligand interactions. *Proteins*, **78**, 1705–1723.

Sirin,S. *et al.* (2016) AB-Bind: Antibody binding mutational database for computational affinity predictions. *Protein Sci*, **25**, 393–409.

Spassov,V.Z. and Yan,L. (2013) pH-selective mutagenesis of protein–protein interfaces: In silico design of therapeutic antibodies with prolonged half-life. *Proteins: Struct., Funct., Bioinform.*, **81**, 704–714.

Teruel,N. *et al.* (2021) Modelling conformational state dynamics and its role on infection for SARS-CoV-2 Spike protein variants. *Plos Comput Biol*, **17**, e1009286.

Wang,R. *et al.* (2005) The PDBbind database: methodologies and updates. *Journal Of Medicinal Chemistry*, **48**, 4111–4119.

Yang,J. *et al.* (2013) BioLiP: a semi-manually curated database for biologically relevant ligand–protein interactions. *Nucleic Acids Res.*, **41**, D1096–D1103.

Yang,Y. and Zhou,Y. (2008) Specific interactions for ab initio folding of protein terminal regions with secondary structures. *Proteins Struct Funct Bioinform*, **72**, 793–803.

Zhang,N. *et al.* (2020) MutaBind2: Predicting the Impacts of Single and Multiple Mutations on Protein-Protein Interactions. *Iscience*, **23**, 100939.

Zhou,H. and Zhou,Y. (2002) Distance-scaled, finite ideal-gas reference state improves structure-derived potentials of mean force for structure selection and stability prediction. *Protein Sci*, **11**, 2714–2726.
